# Relationship between Axial Length and Corneo-Scleral Topography: A Preliminary Study

**DOI:** 10.3390/diagnostics11030542

**Published:** 2021-03-18

**Authors:** Laurent Bataille, Ainhoa Molina-Martín, David P. Piñero

**Affiliations:** 1 Group of Optics and Visual Perception, Department of Optics, Pharmacology and Anatomy, University of Alicante, 03690 Alicante, Spain; bataillebataillebataille@gmail.com (L.B.); ainhoa.molina@ua.es (A.M.-M.); 2 Department of Ophthalmology, Vithas Medimar International Hospital, 03016 Alicante, Spain

**Keywords:** axial length, myopia, corneo-scleral topography, Eye Surface Profiler, profilometry, ocular sagittal height, conjunctiva, biometry

## Abstract

The main objective of the current study was to investigate further the relationship of the overall length of the eye with a great variety of anterior segment parameters, including scleral geometry. A total of 64 eyes of 32 participants with ages from 12 to 52 years were included in this prospective non-randomized single-center study. All participants underwent a complete eye examination, including an analysis of corneo-scleral shape with a Fourier-domain profilometer. A strong negative correlation was found between axial length and temporal-nasal ocular sagittal height difference for different chord lengths. For the right eye, a consistent and stable linear model was obtained to predict the axial length from the spherical equivalent, the corneal diameter, the high-order aberrations root mean square, and the minimum sagittal height for 13- and 14-mm chord. For the left eye, a model was obtained to predict the axial length from the spherical equivalent and the mean corneal curvature, including other parameters such as corneal diameter or high-order aberrations, depending on the chord length, considered for estimating the sagittal height values. More studies with larger samples are needed to confirm these preliminary outcomes.

## 1. Introduction

Most cases of myopia are associated with excessive axial eye growth [1]. Axial myopia is a condition that develops principally during childhood and early adulthood. The excessive elongation of the eye results in blurred distance vision due to images of distant objects focusing in front of the retina. Myopia is defined by a spherical equivalent (SE) lower than −0.5 dioptres (D). Several experimental and clinical studies have clearly demonstrated that both nature (genetics and heredity) and nurture (environment and lifestyle) are important factors in the development of myopia [1]. In the modern lifestyle, the long period of time spent on near-work activities promotes the development of myopia but genetic factors, which are clearly related to the level of susceptibility to these nurture risk factors, have an equivalent influence [2].

At present, myopia is one of the most common visual conditions worldwide [2]. The myopia is described today as an “epidemic” due to the exponential increase in the prevalence of all level of myopia in the last 30 years. The prevalence of myopia is between 10–30% in the adult population in many countries, and 80 to 90% in young adults in some parts of East and Southeast Asia [2]. This visual anomaly is a major public health problem with important challenges. It is necessary to treat a large section of the population with an optical correction in addition to curing the visual damages and blindness associated with pathologic myopia. 

Good visual acuity can be obtained in myopia by prescribing the most optimal optical correction that can be carried out with glasses, contact lenses, or refractive surgery. The risk of adverse ocular tissue changes increases with any level of myopia, but at high levels of myopia (SE worse than −5.0 D) and in pathologic myopia (pathological retinal changes secondary to high myopia), the risk of these effects increases seriously and cause unrecoverable visual impairment such as glaucoma, retinal detachment, and macular holes [3]. According to this, it is important to apply efficient preventive measures to stop or delay the onset of myopia in children, as well as preventive treatments to slow the progression of myopia during childhood.

Several studies have demonstrated geometric anterior sclera changes with increasing level of myopia. Alejandra Consejo et al. demonstrated that the naso-temporal asymmetry of the scleroconjunctival surface decreases with myopia [4]. The more myopic the eye, the less asymmetric the anterior sclera. These authors also found that the anterior scleral shape was well correlated with the axial length and the refractive power. These results agree with the study from Hamed Niyazmanda et al., who demonstrated that there was less nasal-temporal asymmetry of sagittal height and axial radius of curvature of the anterior sclera in high myopes than in emmetropes [5]. This is in agreement with the asymmetric growth of the eye associated with myopia development.

Gökhan Pekel et al. have demonstrated that the anterior scleral thickness was similar in patients with low to moderate myopia and emmetropia [6]. There were no significant correlations between central corneal stromal thickness and anterior scleral thickness in myopic participants. These results contrast with the recent study from Rohit Dhakal et al. that suggested that the use of the anterior scleral thickness might be a biomarker for myopia progression [7]. In their study, Rohit Dhakal at al. demonstrated a significant thinning of the anterior sclera along the inferior meridian with increasing degree of myopia compared with the other three meridians. In this study, no differences in anterior scleral thickness between nasal and temporal meridians were detected. The mean subfoveal posterior scleral thickness for the subset of high myopes was thinner than mean anterior scleral thickness along all the meridians by more than 45%.

Hamed Niyazmanda et al. demonstrated that accommodation and simulated convergence affect the nasal anterior scleral shape, with the greatest changes associated with convergence and being most evident in the more peripheral nasal scleral regions [8]. A significant forward movement of the surface accompanied accommodation, convergence, and their combination. There was flattening with convergence and with the combination of accommodation and convergence. Changes were not significantly different between low to moderate myopes and emmetropes. These results contrast with those from Emily C. Woodman-Pieterse et al. [9], who found a thinning of the anterior temporal sclera during accommodation being more prominent in young adult myopes and increasing with greater accommodation demand. These regional differences may be explained by previously reported regional differences in the ciliary body thickness between refractive groups, and/or regional differences in the biomechanical properties of the sclera between refractive groups.

The main objective of the current study was to investigate the relationship between axial length and corneo-scleral topography, evaluating the level of prediction of the overall length of the eye from the sagittal configuration of the anterior segment. These data would be useful to define new strategies for the prevention and control of myopia based on the detection of factors for prediction of the myopia development that could allow optimizing the currently validated strategies for myopia control and keeping patients with myopia levels suitable for refractive surgery while minimizing the pathological risks associated with excessive elongation of the axial length of the eye.

## 2. Materials and Methods

### 2.1. Patients

A total of 64 eyes of 32 participants (14 males and 18 females) with ages ranging from 12 to 52 years (mean 33 years) were included in this prospective non-randomized single-center study. From these 64 eyes, 34 were myopes, 28 were emmetropes, and 2 were hyperopes. All participants were invited to come to the Optometry Clinic of the University of Alicante where this research was carried out. Both eyes of each subject were evaluated, although some cases were excluded due to the poor-quality measurements. Inclusion criteria were any emmetrope, myope, or hyperope who provided informed consent to participate after an explanation of the study. Healthy eyes with any type or amount of astigmatism or highly irregular sclera were included. Exclusion criteria were any previous ocular surgery which could affect the geometry of the eye, severe dry eye, any active systemic pathology which could affect the ocular tissues, any medication which could affect the physiology of the eye, participants wearing rigid gas permeable contact lenses, and any corneal pathology. Participants wearing soft contact lenses were asked to remove them three days before the measurement. The study methods adhered to the tenets of the Declaration of Helsinki and were approved by the ethics committee of the University of Alicante (Exp UA-2019-08-28). 

### 2.2. Ocular Examination

All participants underwent a complete eye examination including the following tests: case history, measurement of uncorrected and corrected distance visual acuity (CDVA), subjective refraction, slit-lamp biomicroscopy, optical biometry and keratometry with the IOL-Master 500 system (IOLM; Carl Zeiss Meditec AG, Jena, Germany), refractive, aberrometric, topographic, anatomical and tonometry measurements with the VX120 system (Visionix-Luneau Technologies, Chartres, France), and corneoscleral topography analysis with the Fourier domain profilometry-based system Eye Surface Profiler (ESP) (Eaglet-Eye, Houten, The Netherlands). A single experienced examiner (LB) performed all measurements.

The VX120 device is a multi-diagnostic tool which combines Hartmann–Shack refractive and aberrometric measurements, a Placido disk to obtain a topographic analysis, an air-puff system to measure intraocular pressure (IOP), and a Scheimpflug camera to provide anatomical measurements in an automated mode. This device has been described and studied by many authors showing good repeatability for refractive [10,11,12], anatomical [13], corneal, ocular, and internal aberrometric [10,11,14], and corneal topographic parameters [11,12,14].

The IOL-Master 500 system is a partial coherence interferometry biometer considered as a “gold-standard” for intraocular lens power calculations. This is a non-invasive system that measures the distance from the corneal vertex to the retinal pigment epithelium by partial coherence interferometry, being consistently accurate to within ± 0.02 mm or better [15]. Anterior corneal radii are obtained with this system by corneal reconstruction based on reflection images from a 2.5-mm zone, using the index of refraction of 1.3375 to calculate the keratometric power [16].

The Eye Surface Profiler (ESP) is a topography system which offers profiling of the cornea, limbus, and a large portion of sclera, covering an area of up to 20 mm in diameter. For each measurement with the ESP system, the same protocol defined and followed by our research group in previous clinical research was used [17,18]. For the analysis of each eye, the best measurement with a minimum quality index of 95% or more was selected. At least, 15-mm coverage (horizontal diameter) was obtained at each one of the measures. In case 15-mm coverage was not achieved, the measurement was discarded. 

To avoid any interference between VX120, IOLMaster, and ESP measurements, and especially with the instillation of fluorescein required for the measurement with ESP, all the patients first had both eyes evaluated with the VX120 and the IOLMaster, and afterwards with the ESP.

### 2.3. Parameters Evaluated

Different geometric parameters of the corneo-scleral structure have been recorded. The following parameters that were directly extracted from the software of the device (Eaglet ESP Research Edition 3.1.25; Eaglet Eye b.v., Houten, The Netherlands) were considered:

- Inner best-fit sphere (iBFS): best-fit sphere obtained by least squares method of all corneal points from the center to the limbus.
- Limbus best-fit sphere (lBFS): best-fit sphere obtained by least squares method of all points conforming to the limbal area, which is determined as the zone delimited by the first intersection of the inner and outer best-fit spheres with the corneal and scleroconjunctival surfaces at the transition area between conjunctiva and cornea, respectively.
- Outer best-fit sphere (oBFS): best-fit sphere obtained by least squares method of all points from the scleroconjunctival surface detected.
- Mean corneal radius (cr) calculated within the area determined by the limbal diameter.
- Mean scleral radii (sr) calculated for the rest of the area covered by the measurement.
- Sagittal height measurements including mean (MSH), minimum (MinSH), and maximum (MaxSH) sagittal heights. It should be considered that the ESP device calculates the sagittal height as the distance from corneal apex to the line defined by any chord selected.
- Difference between temporal and nasal sagittal heights for a specific chord length (T-NSH).
- For this study, all the sagittal height parameters extracted from the ESP were evaluated for chord lengths of 11, 12, 13, 14, and 15 mm.

The ocular axial length (AL), obtained from the IOLMaster 500 (Carl Zeiss Meditec AG, Jena, Germany), is the distance from the corneal vertex to the retinal pigment epithelium.

The following parameters that were directly extracted from the software of the device VX120 were considered:

- Sphere, cylinder, and spherical equivalent: refraction data at 3-mm pupil diameter.
- Km: average keratometry measurement.
- CCT: central corneal thickness.
- CD: corneal diameter.
- Q: corneal asphericity.
- High order and coma root mean square (RMS) and spherical aberration: ocular aberrations for a 5-mm pupil size.
- IOP: intraocular pressure.

### 2.4. Statistical Analysis

The statistical analysis of the outcomes obtained was performed using the software SPSS v16.0 for Windows (SPSS, Chicago, Illinois, USA). Normality of all data distributions was confirmed by means of the Kolmogorov–Smirnov test. Then, parametric statistics was always applied. Descriptive statistical results were expressed as the mean, standard deviation (SD), median, minimum, and maximum. The partial correlation test was used to assess the degree of association between the ocular axial length and the other parameters recorded, controlling for the effect of the eye (left or right). 

Finally, a multiple linear regression analysis using the backward elimination technique was performed to define a linear model to predict the axial length from anterior segment parameters. Durbin–Watson, tolerance, and variation inflation factor indices were calculated to confirm whether there was collinearity between some parameters and to confirm whether there was not a significant correlation of errors in the regression model. Normality of unstandardized residuals was demonstrated using the Kolmogorov–Smirnov test, which is an elementary condition to accept the model. R^2^ and adjusted R^2^ were used to study the variance explained by the variables of the model.

## 3. Results

This study involved a total of 64 eyes (32 right and 32 left eyes) of 32 participants (14 males and 18 females), with ages ranging from 12 to 52 years (mean 33 years), a mean sphere of −1.58 D (range: −12.00 to 4.00 D) and a mean cylinder of −0.79 D (range: −3.00 to 0.00 D). From these 64 eyes, 34 were myopes, 28 were emmetropes, and 2 were hyperopes. Table 1 shows the descriptive statistics of the main refractive, aberrometric, topographic, biometric, and tonometric characteristics of the eyes included in the study. The descriptive statistics of the main corneoscleral geometric parameters of the eyes included in the study are described in Table 2. All the sagittal height measurements (MSH, T-NSH, MinSH, MaxSH) were obtained for chord lengths increasing from 11 mm to 15 mm.

**Figure 1:**
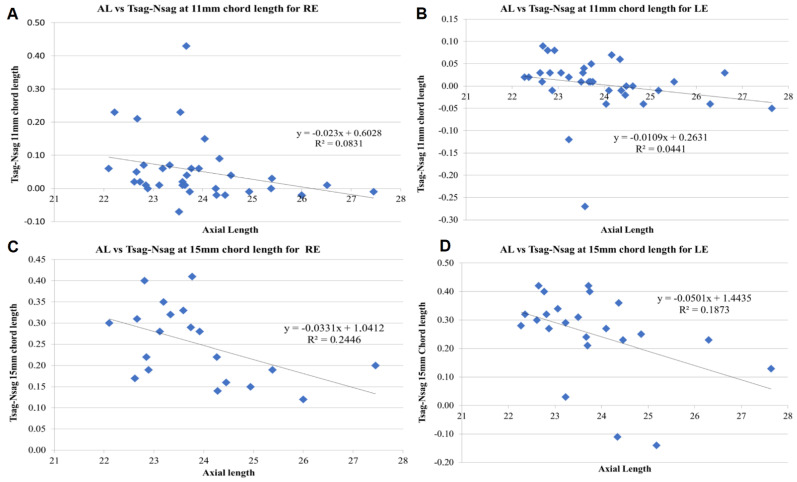
Linear correlation between axial length and the following corneo-topographic data: (**A**) Difference between temporal and nasal sagittal heights (Tsag-Nsag) at 11-mm chord length for the right eye (RE). (**B**) Tsag-Nsag at 11-mm chord length for the left eye (LE). (**C**) Tsag-Nsag at 15-mm chord length for RE. (**D**) Tsag-Nsag at 15-mm chord length for LE. The adjusting line to the data obtained by means of the least-squares fit is shown in each graph.

The partial correlation test controlling for the effect of the eye showed that there were significant correlations between axial length and some geometric data obtained from the Eye Surface Profiler (ESP). Specifically, for chord lengths of 11, 12, 13, 14, and 15 mm, a statistically significant and moderate to strong negative association was found between the axial length and the difference between temporal and nasal sagittal heights (T-NSH) (from *r* = −0.701; *p* < 0.001 for 11-mm chord length to *r* = −0.502; *p* < 0.001 for 15-mm chord length) (Figure 1).

The partial correlation test controlling the effect of the eye of the axial length and the refractive, aberrometric, topographic, biometric and tonometric characteristics obtained with the VX120 system demonstrated a statistically significant and strong correlation between the axial length and the sphere as well as the spherical equivalent (*r* = −0.789 *p* < 0.001 and *r* = −0.786 *p* < 0.001, respectively). A statistically significant but mild to moderate correlation was shown between the axial length and the corneal diameter, the ocular high order aberrations, the ocular coma aberration, and the ocular spherical aberration (*r* = 0.286 *p* = 0.036; *r* = 0.403 *p* = 0.002, *r* = 0.400 *p* = 0.003, and *r* = −0.364 *p* = 0.007, respectively). No correlation between axial length and age, cylinder, mean keratometry, central corneal thickness, corneal asphericity, and intraocular pressure was found.

In this study, a multiple linear regression analysis using the backward elimination technique was performed to define a linear model to predict the axial length from anterior segment parameters. The backward elimination was carried out following the following steps:

1. A significance level of 0.05 was selected to entry in the model.
2. The model was fitted with all possible independent predictors.
3. The predictor with the highest *p*-value was considered. If *p* > 0.10, the predictor was removed.
4. The model was fitted without this last variable and the previous step (3) was repeated until the condition became false.

According to the results of the previous analysis, the following independent variables were considered for this multiple linear regression analysis: axial length (AL) as dependent variable and MinSH, CD, SE, IOP, sr, Km, HOA RMS, and T-NSH as independent variables.

Although both eyes of each subject were evaluated, to avoid the potential interference of the correlation that often exists between the two eyes of the same person, for each chord length, two different multiple linear regression analysis using the backward elimination technique were performed. A first analysis was carried out to define a linear model for the right eye and a second analysis was performed to define a linear model for the left eye. 

Table 3 show the efficiency and validity variables of the right and left eye models to predict the axial length with 13- and 14-mm chord length anterior segment data.

Table 4 and Table 5 show for the right and the left eye at 13-mm and 14-mm chord lengths, respectively, the model coefficients (B) explaining the specific relationship between the parameters of the final model with its standard error (SE) and significance. These tables also show variation inflation factor and tolerance values to assess collinearity relations between parameters. 

In summary, the following linear equations were obtained for predicting the axial length in right and left eyes:

For 13-mm chord length and right eye (*p* < 0.001, adjusted R^2^: 0.866):

(1) AL = 16.50−0.29×SE+0.91×CD+2.31×HOA RMS−1.96×MinSH13

For 13-mm chord length and left eye (*p* < 0.001, adjusted R^2^: 0.700):

(2) AL=37.51 − 0.38 × SE−0.33 × Km

For 14-mm chord length and right eye (*p* < 0.001, adjusted R^2^: 0.843):

(3) AL = 15.62−0.27×SE+0.99×CD+2.77 ×HOA RMS−1.77×MinSH14

For 14-mm chord length and left eye (*p* < 0.001, adjusted R^2^: 0.730):

(4) AL=37.20−0.40×SE−0.33×Km+0.086×CD−3.96×HOA RMS

## 4. Discussion

As shown in Table 1 and Table 2, the main refractive, aberrometric, topographic, biometric, and tonometric characteristics and the main corneoscleral geometric parameters of the eyes included in the study are consistent with those previously reported using the same instruments in healthy eyes [4,5,10,11,12,13,14,16,17,18]. Therefore, the current sample was composed of eyes with normal anterior surface geometry, with no peculiarities introducing bias in the outcomes obtained.

The partial correlation analysis demonstrated a strong negative correlation controlling for the eye (right/left) between axial length and T-NSH at all chord lengths (11 mm to 15 mm). The larger the eye, the lower the temporal-nasal difference was. This confirms that the temporal sagittal height decreases and the nasal sagittal height increases as the axial length becomes longer. Considering that, as expected, a strong positive correlation was present in our study between the axial length and sphere or spherical equivalent, the temporal-nasal asymmetry of the sagittal heights may be a good biomarker of myopic changes. These results are consistent with those obtained in previous studies demonstrating an asymmetric growth of the eye in terms of anterior corneo-scleral geometric data associated with myopia development [4,5]. The more myopic the eye, the less asymmetric was the naso-temporal anterior sclera. This lower asymmetric naso-temporal anterior sclera in myopic eyes could be related to the difference in the insertion of the horizontal rectus muscles. Indeed, for the medial rectus (MR), the length of active muscle is around 37.6 mm with 4-mm tendon length and arc of contact of 6 mm, whereas for the lateral rectus (LR) the length of active muscle is around 36.4 mm, with tendon length 8 mm and arc of contact of 10 mm [19]. As soon as the myopia increases, the axial length of the eye increases, and the tension of the medial and lateral muscles would increase as well. This higher tension and the noticeably less rigid sclera in myopic eyes, compared to healthy eyes [20,21,22], would make the insertion site of the medial and lateral rectus muscles to be displaced backward, as demonstrated by El-Fayoumi D et al. [23]. The mean medial rectus insertion site was 5.47 ± 0.19 mm from the limbus in hyperopes, 5.57 ± 0.18 in emmetropes, and 5.68 ±0.23 mm in myopes, whereas the mean lateral rectus insertion site was 6.81± 0.23 mm from the limbus in hyperopes, 6.89 ± 0.19 in emmetropes, and 7.08 ± 0.16 mm in myopes. These authors reported that the axial length had a moderate positive and significant correlation with the insertional position for the medial and lateral rectus muscles (MR: *r* = 0.417, *p* < 0.001; LR: *r* = 0.410, *p* < 0.001) [23]. The higher tension and the difference of the insertional position of the medial and temporal rectus muscles may explain the decrease in the naso-temporal sagittal height with myopia. As soon as the axial length increases, the medial rectus muscle, which is shorter and inserted closer to the limbus, would create a higher tension closer to the limbus on the nasal side which would increase the nasal sagittal height. As demonstrated by Clark RA. et al., regardless of AL, the globe rotates about a same nasal point and anterior to its geometric center [24] and, consequently, this would suppose that the increased nasal sagittal height would be compensated by a decreased temporal sagittal height to maintain the same rotation axis. This should be investigated further in future studies.

Regarding the linear models to predict the axial length from anterior segment parameters, the results of the multiple linear regression analysis using the backward elimination technique demonstrated a good efficiency and validity of the models for the right eye and left eye at 13- and 14-mm chord length. Future studies should be conducted to evaluate the potential usefulness of these predictions of axial length in eyes with dense ocular optical media in which optical biometry cannot be obtained with reliability. For confirming this, a comparative analysis of predicted axial length measurements with those obtained with ultrasound biometry should be performed. The coefficient of determination, R^2^, demonstrated an acceptable capacity of prediction of the models defined in this study. For the right eye model, the prediction slightly decreased with increasing the diameter of analysis from 89% for a 13-mm chord length to 87.6% for a 14-mm chord length. This decrease may be mainly due to the increasing variability of the measurements obtained with increasing diameter of analysis, as shown in previous studies [17,18]. The right eye model for 13- and 14-mm chord length had the same independent variables (SE, CD, HOARMS, MinSH), indicating that the model was consistent and stable. Regarding the left eye model, it included the independent variables SE and Km for a 14-mm chord, including two additional variables, CD and HOA RMS, when the model was obtained using data for a 13-mm chord. In this case, the eye model showed a higher prediction factor R2 for 14 mm than for 13 mm (0.786 and 0.728, respectively). It should be noted here that the MinSH variable was eliminated in the penultimate step of the backward linear regression analysis for the chord length of 13 mm and in the ultimate step of this analysis for the chord length of 14 mm. Futures studies with larger samples are required to confirm and validate this model, confirming the significance of including this sagittal height variable in the left eye model.

The relation between the axial length and the independent variables defined in the different linear models was consistent with the results of previous research. The association between axial length and spherical equivalent has been demonstrated in several studies [25,26,27,28,29]. In a cross-sectional study with a total of 15,404 European individuals, Tideman et al. demonstrated that the prediction of the axial length from the spherical equivalent (adjusted for age, sex, and height) reached R^2^ = 0.71. The axial length was significantly associated with spherical equivalent, and both variables were associated with visual impairment [28]. The relation between AL, CD, and Km has been investigated extensively by several authors [26,30,31]. The AL significantly correlated with CD and Km [29,30]. Specifically, Km (*r* = –0.444, *p* < 0.001) decreased and CD (*r* = 0.279, *p* < 0.001) increased with elongation of the AL [26]. In longer eyes, higher levels of myopia and flatter cornea are normally present. Lau et al. studied and reported the correlation between ocular higher-order aberrations (HOAs), axial length, and annual axial eye growth after adjusting for other significant predictors of axial length including age, sex, and refractive error in Hong Kong children [32]. According to this study, axial length was greater in subjects with higher myopia (0.32 mm greater per 1 D myopia, *p* < 0.001). Likewise, greater levels of HOA RMS were associated with a longer axial length and a slower annual axial growth rate (0.2 mm longer and 0.1 mm/year decrease per 0.1 μm HOA RMS, respectively, *p* < 0.05). These results demonstrated that HOAs could be an important biomarker to eye growth in a vision-dependent mechanism underlying refractive error development. Regarding the anterior scleral shape (Sagittal Height), Consejo and Rozema recently demonstrated its strong correlation with the axial length (RE and LE: *r* = 0.76, *p* < 0.001), as well as its moderate correlation with the refractive power (LE: *r* = 0.48, RE: *r* = 0.53, both *p* < 0.01) [4]. 

Finally, the linear models to predict the axial length from anterior segment parameters were slightly different for the right and the left eyes. This difference may be related to the axial length asymmetry between eyes. As demonstrated by Rajan et al., the axial length asymmetry between eyes increased with longer axial length (*p* < 0.001). According to this, the axial length asymmetry was expected to be higher in myopic eyes. The axial length difference was 0.5 mm when the axial length for the longer eye was inferior or equal to 22.0 mm, and 4.0 mm when it was superior or equal to 28 mm [33]. This axial length asymmetry has shown the strongest correlation with anisometropia [34].

This study had some limitations that should be acknowledged with regards to the linear models defined to predict the axial length from anterior segment parameters. For the right eye, the linear model was stable and very consistent for 13 and 14-mm chord lengths. However, the left eye model would need more cases to achieve a higher predictability and a better stability for different chord lengths. However, this should be considered as a preliminary study providing information about the potential value of anterior sclera geometry for the prediction of axial length. For this reason, future studies should validate and refine these models in different large samples, including even subjects with different ethnicities. Likewise, more studies are needed to clarify if some corneo-scleral parameters may be act as biomarkers of axial length growth and, therefore, myopia progression.

## 5. Conclusions

There seems to be a correlation among anterior sagittal height measurements of the eye and its axial length. Specifically, differences between nasal and temporal sagittal heights of anterior eye correlates significantly with axial length. Likewise, the axial length might be predicted with acceptable levels of precision by means of a linear equation considering refractive, corneal, and corneo-scleral variables, although the variables implied for the prediction in right and left eyes seem to differ. For the right eye, a consistent and stable model was obtained in the current study to predict the axial length from the spherical equivalent, the corneal diameter, the high-order aberrations root mean square, and the minimum sagittal height for 13- and 14-mm chord. For the left eye, a linear model was also found to predict the axial length from the spherical equivalent and the mean corneal curvature considering corneo-scleral data for a 13-mm chord, whereas another model was defined using corneo-scleral data for a 14-mm chord length to predict the axial length from the spherical equivalent, the mean corneal curvature, the corneal diameter, and the high-order aberrations root mean square. Further studies with a higher number of cases will be necessary to confirm the linear models defined in this study and to improve their stability and consistency.

## Figures and Tables

**Table 1:** Descriptive statistics of the main refractive, aberrometric, topographic, biometric, and tonometric characteristics of the eyes included in the study. Abbreviations: Km, mean keratometry; CCT, central corneal thickness; Q, corneal asphericity; CD, corneal diameter; HOA RMS, ocular high order aberration root mean square; Coma RMS, ocular coma aberration root mean square; IOP, intraocular pressure; AL, ocular axial length.

	Mean	(SD)	Median	Max	Min
Age (years)	33.09	12.86	30.55	51.90	12.01
Sphere (D)	−1.58	2.88	−0.50	4.00	−12.00
Cylinder (D)	−0.79	0.73	−0.50	0.00	−3.00
Spherical equivalent (D)	−1.94	2.94	−0.69	2.50	−13.12
Km (D)	43.63	1.14	43.75	46	41
CCT (µm)	541.85	40.69	542.50	614	438
Q	−0.199	0.161	−0.195	0.210	−0.560
CD (mm)	12.43	0.49	12.42	13.68	11.24
HOA RMS (µm)	0.1932	0.08947	0.1900	0.45	0.05
Coma RMS (µm)	0.1225	0.07724	0.11	0.36	0.01
Spherical aberration (µm)	0.0311	0.07168	0.0300	0.15	−0.17
IOP (mm Hg)	14.26	2.518	14	22	9
AL (mm)	23.88	1.244	23.66	27.64	22.10

**Table 2:** Descriptive statistics of the main corneoscleral geometric characteristics of the eyes included in the study. Abbreviations: iBFS, inner best fit sphere; lBFS, limbus best fit sphere; oBFS, outer best fit sphere; cr, mean corneal radius; sr, mean scleral radius; MSH, mean sagittal height; MinSH, minimum sagittal height; MaxSH, maximum sagittal height; T-NSH, difference between TSH and NSH.

	Mean	(SD)	Median	Max	Min
cr (mm)	8.80	0.270	8.80	9.28	8.22
sr (mm)	13.29	1.588	13.23	20.22	11.17
iBFS (mm)	8.95	0.339	8.92	9.69	7.79
oBFS (mm)	12.50	1.171	12.24	16.70	9.67
lBFS (mm)	6.12	0.432	6.06	7.61	5.10
MSH11 (mm)	1.92	0.090	1.93	2.11	1.56
T-NSH11 (mm)	0.026	0.084	0.015	0.43	−0.27
MinSH11 (mm)	1.765	0.123	1.785	2.01	1.42
MaxSH11 (mm)	1.979	0.087	1.980	2.24	1.61
MSH12 (mm)	2.268	0.123	2.280	2.49	1.80
T-NSH12 (mm)	0.059	0.108	0.050	0.63	−0.14
MinSH12 (mm)	2.117	0.145	2.125	2.42	1.63
MaxSH12 (mm)	2.334	0.112	2.340	2.57	1.81
MSH13 (mm)	2.630	0.132	2.635	2.96	2.11
T-NSH13 (mm)	0.120	0.151	0.100	0.90	−0.10
MinSH13 (mm)	2.482	0.159	2.495	2.79	1.86
MaxSH13 (mm)	2.703	0.110	2.705	3.03	2.50
MSH14 (mm)	2.981	0.151	3.000	3.37	2.40
T-NSH14 (mm)	0.177	0.124	0.180	0.71	−0.12
MinSH14 (mm)	2.857	0.164	2.865	3.11	2.13
MaxSH14 (mm)	3.034	0.206	3.060	3.47	1.89
MSH15 (mm)	3.348	0.173	3.370	3.80	2.71
T-NSH15 (mm)	0.252	0.124	0.280	0.42	−0.14
MinSH15 (mm)	3.236	0.185	3.230	3.58	2.42
MaxSH15 (mm)	3.413	0.159	3.430	3.91	3.06

**Table 3:** Predictability and validity of the right (RE) and left eye (LE) models for predicting the axial length with 13- and 14-mm chord length data. Abbreviations: R^2^ the coefficient of determination R-squared; adjusted R^2^, adjusted R-squared which is a modified version of R-squared that accounts for predictors that are not significant in a regression model; F (Sig) the value of the F-statistic (the corresponding “*p*” value, i.e., the probability of encountering this value, from the F-distribution’s PDF (probability density function)).

		R^2^	Adjusted R^2^	Durbin–Watson	Mean Cook Distance ± SD	F (Sig)	Kolmogorov–Smirnov Test
13-mm chord length	RE model	0.890	0.866	2.172	0.063 ± 0.098	36.465 (*p* < 0.001)	*p* = 0.200
	LE model	0.728	0.700	2.131	0.065 ± 0.105	25.447 (*p* < 0.001)	*p* = 0.200
14-mm chord length	RE model	0.876	0.843	1.970	0.070 ± 0.083	26.513 (*p* < 0.001)	*p* = 0.143
	LE model	0.786	0.730	1.884	0.809 ± 3.447	13.813 (*p* < 0.001)	*p* = 0.200

**Table 4:** Summary of the right (RE) and left eye (LE) models for predicting the axial length with 13-mm chord length data. Abbreviations: B, model coefficient; SE, standard error; Sig, significance; VIF, variance inflation factor.

		Predictors	B	SE	Beta	t	Sig.	Tolerance	VIF
**13-mm chord length**	RE model	SE	−0.291	0.035	−0.712	−8.216	<0.001	0.812	1.232
		CD	0.906	0.148	0.480	6.137	<0.001	0.999	1.001
		HOA RMS	2.311	1.016	0.211	2.274	0.035	0.710	1.408
		MinSH13	−1.959	0.619	−0.267	−3.166	0.005	0.861	1.162
	LE model	SE	−0.384	0.057	−0.821	−6.787	<0.001	0.977	1.023
		Km	−0.330	0.103	−0.386	−3.194	0.005	0.977	1.023

**Table 5:** Summary of the right (RE) and left eye (LE) models for predicting the axial length with 14-mm chord length data. Abbreviations: B, model coefficient; SE, standard error; Sig, significance; VIF, variance inflation factor.

		Predictors	B	SE	Beta	t	Sig.	Tolerance	VIF
**14 m chord length**	RE model	SE	−0.266	0.042	−0.642	−6.344	0.000	0.807	1.239
		CD	0.989	0.168	0.554	5.895	0.000	0.934	1.070
		HOA RMS	2.774	1.121	0.280	2.475	0.026	0.646	1.547
		MinSH14	−1.774	0.708	-0.270	−2.508	0.024	0.715	1.399
	LE model	SE	−0.399	0.058	−0.977	−6.920	0.000	0.715	1.400
		Km	−0.329	0.090	−0.476	−3.672	0.002	0.845	1.183
		CD	0.086	0.047	0.231	1.852	0.084	0.912	1.097
		HOA RMS	−3.964	1.475	−0.380	−2.687	0.017	0.711	1.407

## Data Availability

Data available on request from the authors.
